# Evaluation of three commercial kits for mycoplasma NAT assays: selection and quality improvement

**DOI:** 10.1186/1753-6561-5-S8-P127

**Published:** 2011-11-22

**Authors:** Fabien Dorange, Frédérick Le Goff, Nicolas Dumey

**Affiliations:** 1 Texcell, Evry, France, 91058

## Background

Mycoplasma testing on cell lines or biological products used to be performed based on classical methods such as agar and broth medium and/or indicator cell culture. However, these methods require a long incubation period and are not adapted for samples, like live-attenuated vaccine viruses (which can not completely be neutralized) or cell therapy products (with short shelf life). NAT assays have several advantages including rapid-time to results, robustness and sensitivity. The European Pharmacopoeia updated the 2.6.7 section by adding the detection of Mycoplasma with NAT methods as an alternative to one or both classical methods.

Texcell’s offers for Mycoplasma testing, which already included classical methods, were incremended with NAT assay. For this purpose, 3 commercial kits based on NAT assay were evaluated based of their claim to meet the European Pharmacopeia guidance for nucleic acid amplification techniques for Mycoplasma testing, including sensitivity and range of detection: CytoCheck® from Greiner, MycoTOOL® from Roche, MycoSEQ® Mycoplasma detection kit from Life Technologies.

## Material and methods

5 mycoplasma species were chosen among the 9 strains listed in the European Pharmacopeia.

*Mycoplasma Pneumoniae*: CIP 103766T

*Mycoplasma Hyorhinis*: ATCC 179891

*Acholeplasma Laidlawii*: ATCC 23206

*Mycoplasma Orale*: provided by Greiner Bio-One

*Mycoplasma Synoviae*: provided by Greiner Bio-One

1 ml of different concentrations of mycoplasma were tested according to the supplier’s instructions.

## Results

For the mycotool results, none of the mycoplasma species tested were detected above the required limit of detection of 10 cfu/ml. Investigations showed that quantity of nucleic acids in our experimental design was too small to be efficiently recovered with the precipitation based extraction procedure. Indeed, the mycoTOOL**®** kit was designed to be used in conjonction with CHO cells (5x10^6^ cells/ml), acting like carrier DNA for the precipitation step. Since this study, the kit’s supplier has included a carrier DNA for samples containing low level of nucleic acid.

Both remaining selected kits (MycoSEQ® and CytoCheck®) met the sensitivity parameters and were compared (Table 1).

**Table 1:** Comparison of the MycoSEQ® Mycoplasma detection kit and CytoCheck® kit.

	KIT supplier		
Parameters	MycoSEQ®	CytoCheck®	Priority
	Applied Biosystems	Greiner	
tested sensitivity	<10 CFU/ml	<10 CFU/ml	1
Species coverage	> 90	41 identified	2
Time to results	Same day	Same day	3
Contamination prevention	No post-amplification sample handling	post-amplification sample handling	4
Reagent price ratio per test	1	10	5
Equipment	Real-Time PCR 7500	PCR machine scanner	6
quantitation	yes	no	7

Although these kits use a different technology, similar results (sensitivity, range of detection) were obtained. The lower sensitivity observed with the MycoSEQ® kit for *M. Pneumoniae* is explained by the presence of non-viable mycoplasmas induced during thawing/freezing cycle for stocks preparation.

According to its safer use (no post-amplification handling), its lower cost and quantitation possibilities, the MycoSEQ® kit was preferred.

Performance validation was conducted in Texcell facilities using the MycoSEQ® Mycoplasma detection kit.

## Conclusion: selection of a high performance kit and quality improvement

Although two of the selected kits show similar results, the MycoSEQ® kit was chosen for its lower risk of cross contamination. However, all the tested kits (including the selected one) suffer from a lack of appropriate extraction controls. Therefore, tests performed systematically at Texcell include viable mycoplasmas (positive and inhibitory controls). This Texcell’s added value improves the quality of the whole process from the first concentration step (centrifugation) to the qPCR results. At Texcell, in 2010, the first samples were tested with success in a “GMP regulation context”.

